# *Ficus carica* L. (Fig) promotes nerve regeneration in a mouse model of sciatic nerve crush

**DOI:** 10.1002/2211-5463.13859

**Published:** 2024-06-28

**Authors:** Satoshi Sugita, Kotaro Tamura, Kohjiro Hashizume, Yoshihiko Minegishi, Noriyasu Ota

**Affiliations:** ^1^ Biological Science Research Kao Corporation Tochigi Japan

**Keywords:** *Ficus carica* L., fig, macrophage, nerve regeneration, peripheral nerve injury, phytochemical

## Abstract

Peripheral nerve injuries result in significant loss of motor and sensory function, and the slow rate of nerve regeneration can prolong recovery time. Thus, approaches that promote axonal regeneration are critical to improve the outcomes for patients with peripheral nerve injuries. In this study, we investigated the effects of *Ficus carica* L. (fig) and *Vaccinium macrocarpon* Ait. (cranberry), which are rich in phytochemicals with demonstrable and diverse medicinal properties, on nerve regeneration in a mouse model of sciatic nerve crush. Our investigation revealed that fig extract, but not cranberry extract, prevented the decline in muscle weight and nerve conduction velocity induced by nerve crush. The fig extract also mitigated motor function impairment, myelin thinning, and axon diameter reduction, indicating its potential to promote nerve regeneration. Furthermore, the fig extract enhanced macrophage infiltration into the nerve tissue, suggesting that it could ameliorate nerve injury by promoting tissue repair via increased macrophage infiltration. The study provides valuable insights into the potential of the fig extract as a novel agent promoting nerve regeneration. Further investigation into the mechanisms underlying the action of fig extracts is needed to translate these findings into clinical applications for patients with peripheral nerve injuries.

## Graphical abstract

*Ficus carica* L. (fig) is rich in phytochemicals with demonstrable and diverse medicinal properties. The results of this study demonstrated that the fig extract promoted nerve regeneration and enhanced macrophage infiltration into the nerve tissue induced by nerve crush injury in mice, suggesting that the fig extract could ameliorate nerve injury by promoting tissue repair via increased macrophage infiltration.

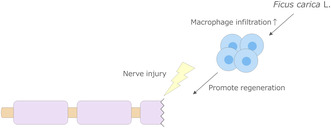

**Abbreviations**
BDNF: brain‐derived neurotrophic factor
CNTF: ciliary neurotrophic factor
Cranberry: *Vaccinium macrocarpon* Ait
Fig: *Ficus carica* L.
MNCV: motor nerve conduction velocity
NGF: nerve growth factor

## Introduction

Peripheral nerve injury is a devastating condition that causes loss of motor and sensory function. While the peripheral nerves are innately capable of regeneration, the rate of regeneration is slow, approximately 1 mm per day [1]. This slow pace becomes particularly pronounced when the injury occurs at a substantial distance from the innervated site, prolonging recovery by months or even years. Approaches that promote axonal regeneration are needed since delayed repair has a negative impact on satisfactory sensory and motor recovery [2].

In the event of peripheral nerve injury, Wallerian degeneration occurs, leading to the degeneration of axons and myelin distal to the site of injury [3], resulting in atrophy of their innervating muscles [4]. Schwann cells and macrophages play a pivotal role in nerve regeneration. Following injury, Schwann cells rapidly dedifferentiate and proliferate within the basal lamina tube [5, 6]. In the process, myelin genes including myelin basic protein (MBP), myelin protein zero (MPZ), and myelin‐associated glycoprotein (MAG) are repressed [7]. Then, during the repair process, Schwann cells promote axonal growth via the release of neurotrophic factors such as the brain‐derived neurotrophic factor (BDNF), nerve growth factor (NGF), and ciliary neurotrophic factor (CNTF) [8]. In fact, administration of these neurotrophic factors after nerve injury has been shown to promote nerve regeneration [9, 10, 11, 12]. In addition to secreting neurotrophic factors, Schwann cells produce chemokines that recruit immune cells, primarily macrophages [13]. Macrophages recruited to the injury site play an essential role in nerve regeneration by eliminating myelin residues and promoting angiogenesis [14, 15]. Depletion of CD11b‐positive macrophages in mice resulted in reduced myelin debris clearance and impaired axon regeneration and functional recovery [16]. Therefore, these findings support the notion that promoting the secretion of neurotrophic factors and increasing macrophage infiltration contribute to peripheral nerve regeneration.

Phytochemicals such as polyphenols, organosulfur compounds, alkaloids, and terpenoids are plant‐derived compounds found in fruits and vegetables [17]. They are known to possess a variety of medicinal properties, including anti‐inflammatory and anticancer effects [18], while some have been shown to promote nerve regeneration [19, 20]. *Ficus carica* L. (fig) and *Vaccinium macrocarpon* Ait. (cranberry), widely consumed as dried fruits, are rich in phytochemicals such as anthocyanins and flavonoids [21, 22] that exhibit various health benefits, including antioxidant and anti‐inflammatory effects [23, 24, 25, 26]. Although these fruits have long been used for health care applications owing to their medicinal properties [21, 22], no study has reported on their effectiveness in promoting nerve regeneration.

In this study, we investigated the effects of fig and cranberry extracts on nerve regeneration in a mouse model of sciatic nerve crush and elucidated their effect on neurotrophic factors and macrophage infiltration.

## Materials and methods

### Preparation of fig and cranberry extracts

In this experiment, 500 g of dried fig or dried cranberry each was divided into quarters and soaked in 5 L of 99.5% ethanol for 8 days at room temperature, respectively. After filtration, the extracts were concentrated under reduced pressure. Thereafter, the concentrates were diluted with ion‐exchanged water and lyophilized to yield 99.5 g of dried fig extract and 133.2 g of dried cranberry extract.

### Animals

C57BL/6J mice were purchased from Charles River Laboratories, Japan, and maintained at 23 ± 2 °C under a 12 h/12 h light–dark cycle. All animal experiments were approved by the Animal Care Committee of Kao Corporation (approval number: S17137‐0000, S18057‐0000) and conducted in accordance with the committee's Guidelines for the Care and Use of Laboratory Animals.

Male mice (9 weeks old) with similar body weights were divided into the following four groups: sham operation group (sham), control diet (control), fig extract diet (fig), and cranberry extract diet (cranberry). For experiments to study the effects on motor function, male mice (9 weeks old) with similar body weights were divided into the following three groups: sham, control, fig. The control diet contained 10% fat (w/w), 20% casein, 55.5% potato starch, 8.1% cellulose, 4% minerals, 2.2% vitamins, and 0.2% methionine. In the fig and cranberry groups, fig or cranberry extract was added into the control diet, so that the proportion of each extract was 1%, necessitating adjustment of the potato starch content to 54.5%. All animals were fed *ad libitum* on their respective diets from 1 week before to 4 weeks after nerve crush injury and then sacrificed under isoflurane (Abbott Laboratories, Chicago, IL, USA) anesthesia.

### Nerve crush

At 10 weeks of age, the mice were anesthetized with isoflurane to expose the right sciatic nerve. The nerve was compressed tightly with tweezers at the mid‐thigh level for 10 s, and the incision was sutured. In the sham group, the right sciatic nerve was exposed, and the incision was sutured. For experiments to study the effects on motor function, at 10 weeks of age, the mice were anesthetized with isoflurane to expose the sciatic nerves in both legs. The nerves were compressed tightly with tweezers at the mid‐thigh level for 10 s, and the incisions were sutured. In the sham group, the sciatic nerves of both legs were exposed and the incisions were sutured.

### Motor nerve conduction velocity

The mice were anesthetized with isoflurane and placed on a heat‐retaining pad to maintain the body temperature at 37 °C. The motor nerve conduction velocity (MNCV) was determined using established methods [27, 28, 29, 30]. In brief, the right sciatic‐tibial nerve was electrically stimulated using needle electrodes (MEB9402‐MB; Nihon Kohden, Tokyo, Japan) at the ankle and at the sciatic notch, and M‐waves were recorded from the second interosseous muscle of the foot. The distance between the two stimulation sites was divided by the latency difference.

### Hanging test

To examine motor function, an inverted grid hanging test was performed [31]. The mice were placed on the center of a grid of wire mesh placed 25 cm above the table and the wire mesh was slowly inverted. The time stayed before falling was measured as a maximum of 600 s. Each mouse was tested five times, and the average of the top two values was calculated. At least 10 min were allowed between measurement.

### Nerve histology

To visualize the nerve structures, the peroneal nerve was pre‐fixed with 2.5% glutaraldehyde overnight at 4 °C, and post‐fixed with 1% osmium tetroxide for 2 h at 4 °C. After embedding in EPON resin (TAAB Laboratories Equipment Ltd, Berks, England), the specimens were sectioned into 1.5‐μm thick slices and stained with 0.5% toluidine blue. Bright‐field images of the sectioned specimens were acquired using an all‐in‐one fluorescence microscope (BZ‐X700; Keyence, Osaka, Japan) at a magnification of 100×. The g‐ratio (i.e., the ratio between the inner and outer diameters of the myelin sheath) was determined for more than 100 axons per section, which were randomly selected with the help of a grid (the vertex of a 150 μm^2^ square) using ImageJ software (National Institutes of Health). Axon diameter was measured as more than 100 axon diameters per section.

For F4/80 immunostaining, the peroneal nerve was fixed with 4% paraformaldehyde in phosphate‐buffered saline (Fujifilm‐wako, Osaka, Japan) and embedded in paraffin. Three‐micrometer‐thick sections of the peroneal nerve were stained using anti‐mouse F4/80 antibody (#MCA497GA, Bio‐Rad Laboratories, Hercules, CA, USA) [32]. The number of nuclei surrounded by F4/80‐positive cells in two cross‐sections was counted and expressed as the mean value per millimeter squared.

### Quantitative reverse transcription polymerase chain reaction

Total RNA was extracted from the tibial nerve using the RNeasy Plus Universal Mini Kit (QIAGEN, Hilden, Germany), as described in the manufacturer's protocol. Quantitative reverse transcription polymerase chain reaction (PCR) was performed using a TaqMan probe (Thermo Fisher Scientific, Waltham, MA, USA) on an ViiA 7 Real‐Time PCR System (Thermo Fisher Scientific). For myelin genes, MPZ (#Mm00485139_m1), MBP (#Mm01266402_m1), and MAG (#Mm00487538_m1) were measured; for neurotrophic factors, BDNF (#Mm04230607_s1), NGF (#Mm00443039_m1), and CNTF (#Mm00446373_m1) were measured; and for macrophage‐related genes, F4/80 (#Mm00802529_m1, M1 and M2 marker), CD86 (#Mm0044540_m1, M1 marker), and CD206 (#Mm01329362_m1, M2 marker) were measured. Data were normalized to the acidic ribosomal protein P0 (Rplp0) mRNA content.

### Statistical analyses

All values were presented as the mean ± standard error (SEM). Statistical analysis was performed using the analysis of variance, followed by Dunnett's test (SPSS software version 24; IBM, Armonk, NY, USA). Statistical significance was set at *P* < 0.05.

## Results

### Effects of fig and cranberry extracts on body weight, muscle weight, and MNCV in nerve crush injury

**Fig. 1:**
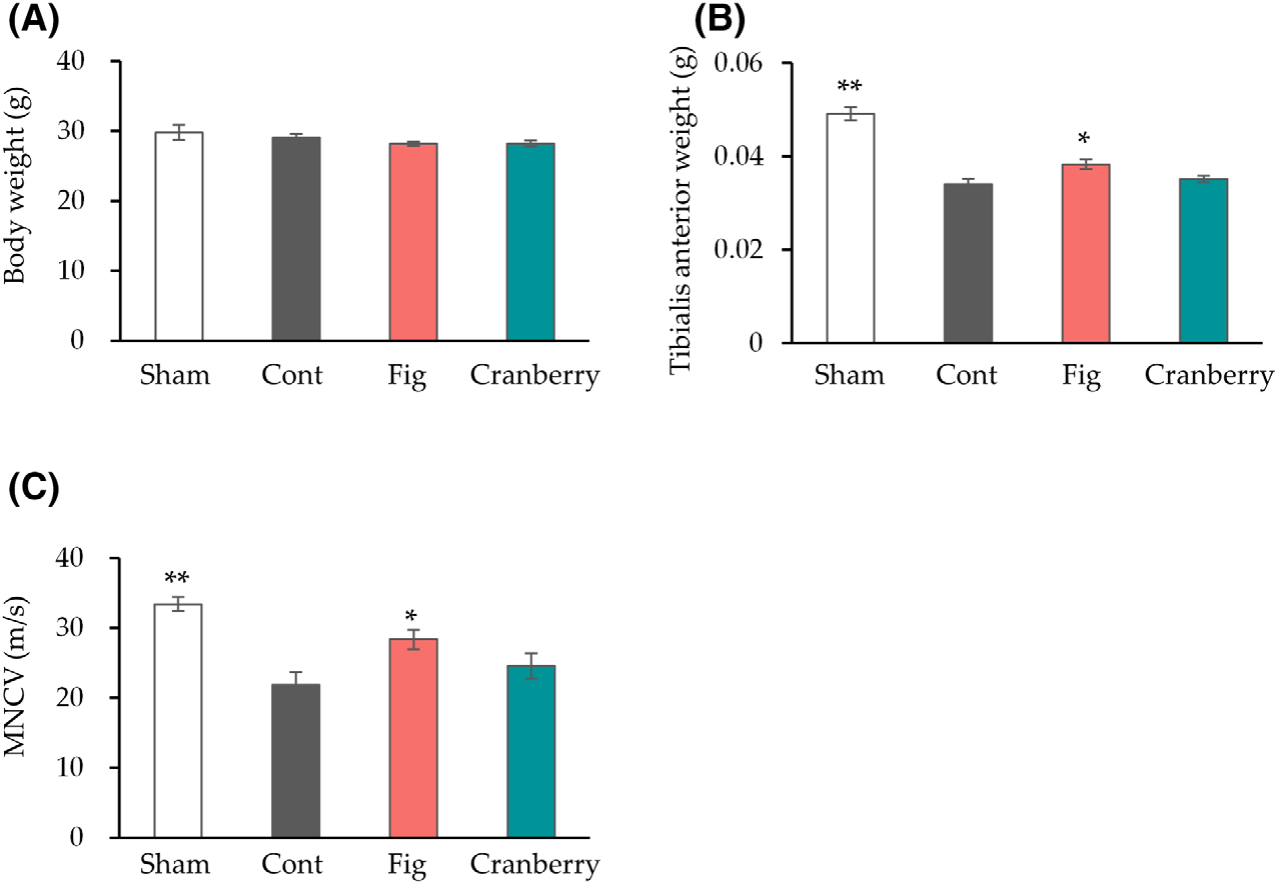
The effect of fig and cranberry extracts on nerve crush injury. Body weight (A) and tibialis anterior muscle weight (B) at 4 weeks after sciatic nerve crush (*n* = 6 in each group). (C) Motor nerve conduction velocity (MNCV) at 4 weeks after sciatic nerve crush was analyzed (*n* = 7 in Sham, *n* = 6 in Cont, *n* = 8 in Fig and Cranberry group). Values are presented as the mean ± SEM. *P < 0.05, **P < 0.01 vs. Cont by Dunnett's test. Cont, control diet group; Cranberry, cranberry extract diet group; Fig, fig extract diet group; Sham, sham operation group.

The mice from all groups were fed their respective diets *ad libitum* for 1 week before the induction of nerve crush. Nerve crush injury was induced in all groups but the sham group to determine the potential of fig and cranberry extracts in promoting nerve regeneration. MNCV is an electrophysiological parameter used to assess the functional recovery of peripheral nerves after nerve crush injury [33, 34]. Nerve crush injury causes atrophy of the innervating muscles and a decrease in MNCV [4, 33]. The body weight, tibialis anterior muscle weight, and MNCV were measured 4 weeks after nerve crush (Fig. 1). The body weight in the sham, fig, or cranberry group did not differ significantly from that in the control group. The control group exhibited a significant decrease in tibialis anterior muscle weight, the sciatic nerve innervating muscle, and MNCV compared to the sham group. While the tibialis anterior muscle weight and MNCV did not differ significantly between the cranberry and control groups, the fig group showed a significant increase in both parameters. These results suggest that fig consumption inhibits nerve injury‐induced decrease in the tibialis anterior muscle weight and MNCV.

### Effects of fig extract on motor function and nerve structure in nerve crush injury

**Fig. 2:**
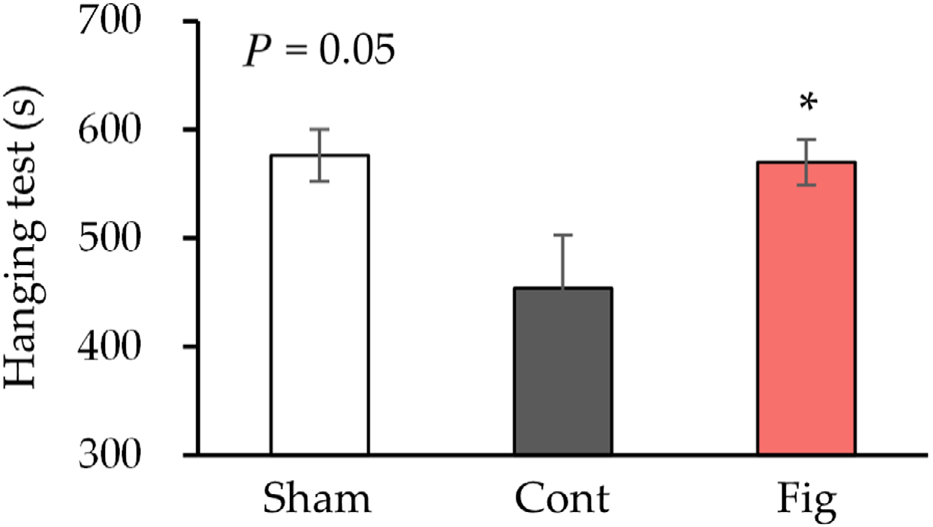
Motor function after 4 weeks of sciatic nerve crush. Hanging test at 4 weeks after bilateral sciatic nerve crush was conducted (*n* = 6 in Sham, *n* = 8 in Cont, *n* = 8 in Fig group). Values are presented as the mean ± SEM. *P < 0.05 vs. Cont by Dunnett's test. Cont, control diet group; Fig, fig extract diet group; Sham, sham operation group.

To investigate the effect of fig extract on the inhibition of nerve injury, the effect on motor function was examined by a hanging test (Fig. 2). Previous studies have reported that crushing one leg does not lead to a decline in motor function after 4 weeks [34], whereas bilateral sciatic nerve crush injury has been reported to decrease motor function [35]. Therefore, in this study, the sciatic nerves of both hindlimbs were crushed, and motor function was evaluated. The control group exhibited a trend of decreased hanging time compared to the sham group (*P* = 0.05). In contrast, the fig group showed a significant increase in hanging time compared to the control group.

**Fig. 3:**
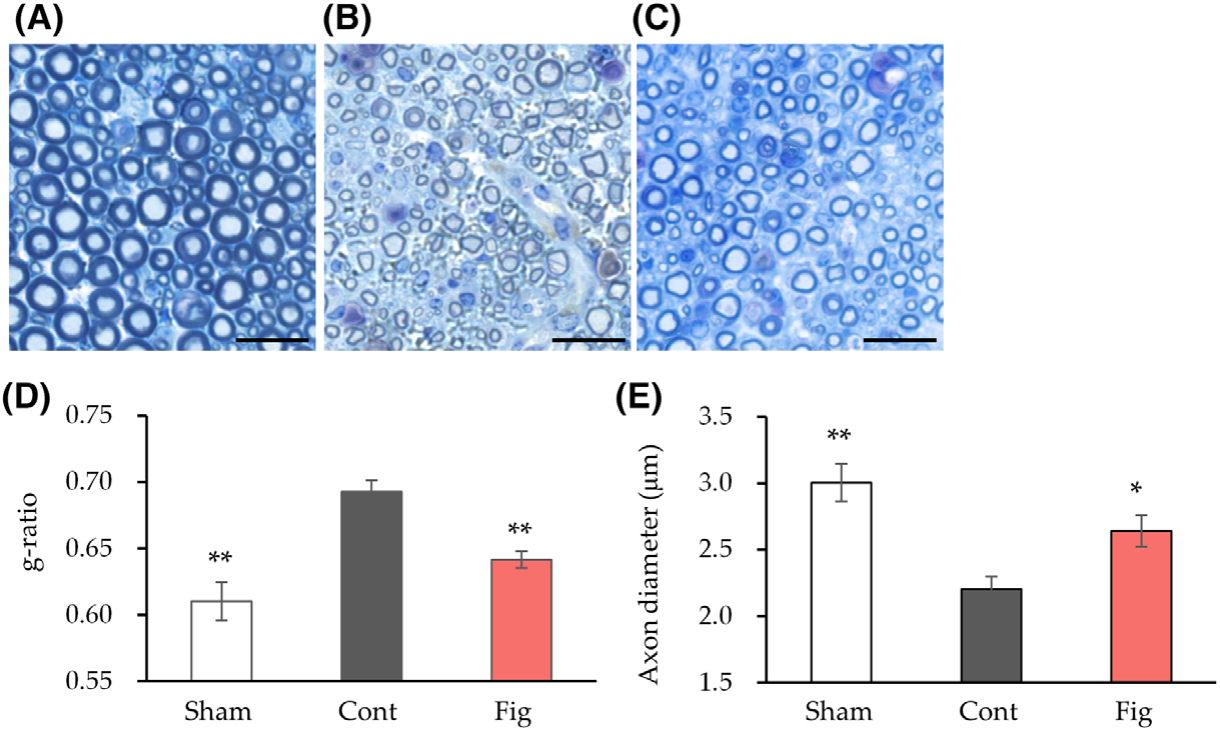
Histological analysis of nerve tissue after 4 weeks of sciatic nerve crush. (A–C) Semi‐thin cross‐sections of the peroneal nerve stained with toluidine blue. Images of nerves at 4 weeks after sciatic nerve crush are shown (A, Sham; B, Cont; C, Fig). (D) The ratio of nerve fiber diameter divided by axon diameter (g‐ratio) was calculated (*n* = 3 in Sham and Cont, *n* = 6 in Fig group). (E) Axon diameter was calculated (*n* = 3 in Sham, *n* = 6 in Cont, *n* = 6 in Fig group). Values are presented as the mean ± SEM. *P < 0.05, **P < 0.01 vs. Cont by Dunnett's test. Scale bars: 20 μm. Cont, control diet group; Fig, fig extract diet group; Sham, sham operation group.

Histological analysis was conducted to further investigate the inhibitory effect of fig extract on nerve injury after 4 weeks (Fig. 3). The control group exhibited marked myelin thinning and a significant increase in the g‐ratio, an indicator of myelin thickness, compared to the sham group (Fig. 3A–D). In addition, there was a significant decrease in axon diameter compared to the sham group (Fig. 3E). In contrast, the fig group showed a significant decrease in the g‐ratio and a significant increase in axon diameter compared to the control group.

These results show that fig extract inhibits motor deficits, myelin thinning, and axon diameter reduction caused by nerve injury, suggesting that figs promote nerve regeneration.

### Effect of fig extract on neurotrophic and macrophage infiltration in nerve crush injury

**Fig. 4:**
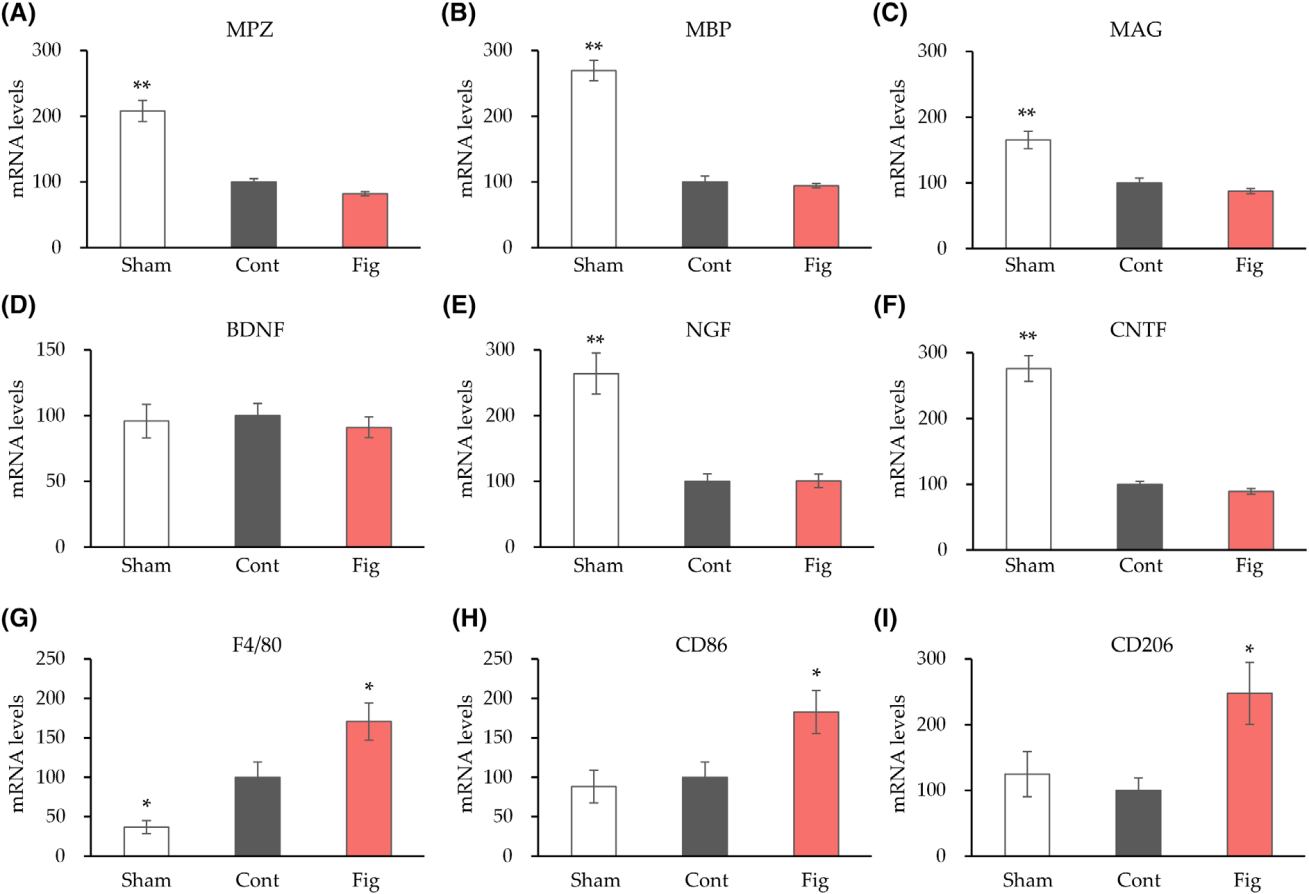
mRNA expression changes after 4 weeks of sciatic nerve crush. Tibial nerve gene expression changes for myelin genes (MAG, myelin‐associated glycoprotein; MBP, myelin basic protein; MPZ, myelin protein zero) (A–C), neurotrophic factors (BDNF, brain‐derived neurotrophic factor; CNTF, ciliary neurotrophic factor; NGF, nerve growth factor) (D–F), macrophage‐related gene expression (F4/80, CD86, CD206) (G–I) were measured. Values are presented as the mean ± SEM (*n* = 6 in each group). *P < 0.05, **P < 0.01 vs. Cont by Dunnett's test. Cont, control diet group; Fig, fig extract diet group; Sham, sham operation group.

The small amount of neural tissue limited our analysis. As a result, our strategy was to initially identify the relevant mechanisms among the wide range of mechanisms involved in nerve regeneration, followed by a more detailed analysis. We first performed a gene expression analysis, which can measure a large number of genes at a small amount. We analyzed myelin genes (MPZ, MBP, MAG), neurotrophic factors (BDNF, NGF, CNTF), and macrophage‐related gene expression (F4/80, CD86, CD206) to elucidate the mechanism underlying the inhibitory effect of fig extract on nerve injury (Fig. 4). There were no significant differences in myelin gene (MPZ, MBP, MAG) and neurotrophic factors (BDNF, NGF, CNTF) gene expression in the fig group compared to the control group. For macrophage‐related gene expression, the control group showed a significant increase in F4/80 gene expression compared to the sham group. On the other hand, the fig group showed significantly increased gene expression of F4/80, CD86, and CD206 compared to the control group.

**Fig. 5:**
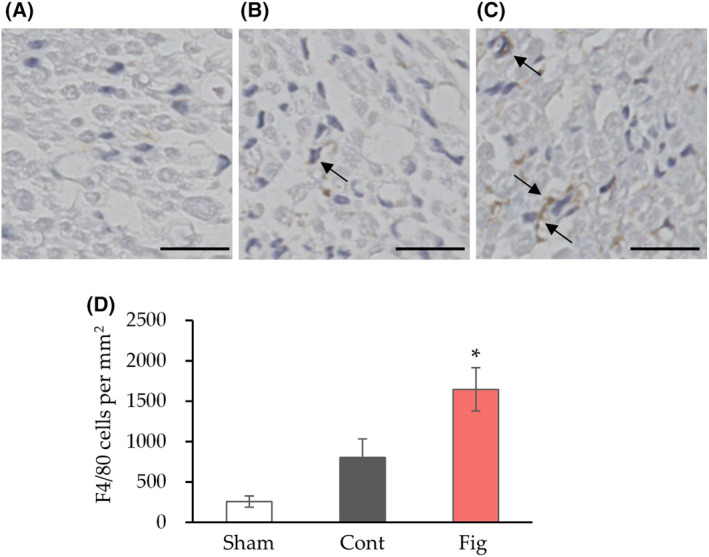
The effect of fig extract on macrophage infiltration in nerve crush. (A–C) Representative F4/80 immunostaining images in cross section of the peroneal nerve (A, Sham; B, Cont; C, Fig, arrows show positive cells) and quantitative results of the number of F4/80 positive cells (D) were measured 4 weeks after sciatic nerve crush. Values are presented as the mean ± SEM (*n* = 6 in Sham and Cont, *n* = 5 in Fig group). *P < 0.05 vs. Cont by Dunnett's test. Scale bars: 20 μm. Cont, control diet group; Fig, fig extract diet group; Sham, sham operation group.

F4/80 immunostaining has been well documented for macrophage infiltration after nerve crush [36, 37]. We performed immunostaining of F4/80 to confirm the effect of fig extract on macrophage infiltration (Fig. 5). Consistent with the gene expression analysis, the number of F4/80 positive cells was significantly higher in the fig group compared to the control group. These results suggest that fig extract could ameliorate nerve injury by promoting tissue repair by augmenting macrophage infiltration.

## Discussion

We examined the impact of fig and cranberry extracts, both rich in phytochemicals, on nerve regeneration. Our findings revealed that only fig extract prevented a decline in muscle weight and nerve conduction velocity induced by nerve injury. Furthermore, fig extract was shown to alleviate the loss of motor function, myelin thinning, axon diameter reduction caused by nerve injury, indicating its potential to promote nerve regeneration. In addition, fig extract enhanced macrophage infiltration, suggesting that fig extract could ameliorate nerve injury by promoting tissue repair via augmented macrophage infiltration.

In the present study, we found that fig extract possesses the ability to promote nerve regeneration. The fig, an important member of the genus Ficus belonging to the Moraceae family, is thought to be the first domesticated plant in the world, as it was discovered at the early Neolithic Gilgal I site, which dates approximately 11 400 years ago [38]. The myriad actions of figs include antioxidant, anti‐inflammatory, and anti‐diabetic effects [39, 40, 41, 42, 43]. However, their ability to promote nerve regeneration has not been reported. Recently, fig extract was reported to inhibit denervation‐induced muscle atrophy [44]. However, that study did not examine the effect of fig extract on nerves and proposed that the suppression of inflammation in the muscles located downstream of the nerve contributed to the inhibition of muscle atrophy. In this study, we showed that fig extract affects nerves because it enhances macrophage infiltration into nerve tissue and suppresses myelin thinning. Therefore, it can be postulated that the suppression of muscle atrophy induced by denervation is an indirect result of the effect on the nerves. Future *in vitro* experiments are required to determine whether fig extract exerts effects on muscles in addition to nerves. The active components responsible for the nerve regenerative effect of fig extract are unknown. Figs contain various phytochemicals, including the flavonoids rutin and quercetin, the coumarins psoralen and isopsoralen, and the anthocyanin cyanidin‐3‐O‐rhamnoglucoside [45]. In order to identify the active components, it is necessary to fractionate the fig extract and conduct screening. We endeavor to establish an appropriate screening system in the future, to identify the active components responsible for the nerve regenerative effect of fig extract.

Macrophages recruited after nerve injury persist in the nerve for at least one month [46] and contribute to nerve regeneration by eliminating myelin residues and promoting angiogenesis [14, 15]. In the present study, fig extract enhanced the expression of macrophage‐related genes such as F4/80, CD86, and CD206, and increased the number of F4/80‐positive cells, suggesting that fig extract could ameliorate nerve injury by promoting tissue repair through increased macrophage infiltration. Macrophages are classified into M1 and M2 macrophages according to the Th1/Th2 dichotomy [47, 48, 49]. M1 macrophages promote inflammation and contribute to myelin residue removal, while M2 macrophages produce anti‐inflammatory cytokines and contribute to nerve regeneration, including angiogenesis and matrix remodeling [3, 48, 49, 50, 51]. Previous studies have shown that accelerating the polarization of macrophages from the M1 to M2 phenotype promotes nerve regeneration [52], and that deficiency of IL‐10, a key factor involved in the transition of M2 macrophages, impairs nerve regeneration [53], suggesting that an efficient transition from the M1 to M2 phenotype is crucial for nerve regeneration. In this study, the M1 and M2 marker F4/80 was increased, as was the M2 marker CD206, suggesting that elevated M2 macrophage levels may have promoted nerve regeneration. Further studies should investigate the effects of figs on M1 and M2 macrophages in detail using fluorescence‐activated cell sorting.

Peripheral nerve injury significantly diminishes patients' quality of life, necessitating the development of techniques to promote nerve regeneration from the medical perspective. Clinically, nerve autograft implantation, which entails transplantation of healthy nerve, and the use of hollow conduits to bridge the nerve, are utilized to treat injured nerves [54, 55]. However, these methods are beset by several limitations, particularly in cases of large‐gap injuries that do not recover adequately. In the present study, we demonstrated that the ingestion of fig extract promotes nerve regeneration. In the future, we will further investigate the potential of figs for nerve regeneration and pursue an in‐depth understanding of the mechanism of nerve regeneration, with the ultimate goal of clinical application.

## Conflict of interest

All authors are employees of Kao Corporation.

### Peer review

The peer review history for this article is available at https://www.webofscience.com/api/gateway/wos/peer‐review/10.1002/2211‐5463.13859.

## Author contributions

SS, YM, and NO contributed to the conceptualization. SS, KT, and KH contributed to the investigation and methodology. SS contributed to the project administration and writing—original draft. YM and NO contributed to the supervision. KT, KH, YM, and NO contributed to the writing—review and editing. All authors have read and agreed to the published version of the manuscript.

## Data Availability

The data that support the findings of this study are available from the corresponding author upon reasonable request.
